# Icariin ameliorates oxidative stress-induced inflammation, apoptosis, and heart failure in isoproterenol-challenged Wistar rats

**DOI:** 10.22038/IJBMS.2023.66481.14589

**Published:** 2023

**Authors:** Sumit Sharma, Ashif Iqubal, Vasim Khan, Kalicharan Sharma, Abul Kalam Najmi, Syed Ehtaishamul Haque

**Affiliations:** 1 Department of Pharmacology, School of Pharmacy Education and Research, Jamia Hamdard, New Delhi – 110062, India; 2 Department of Pharmaceutical Chemistry, Delhi Pharmaceutical Sciences and Research University, New Delhi - 110017, India

**Keywords:** Epimedium, Heart failure, NF-kappaB, Oxidative stress, Sildenafil

## Abstract

**Objective(s):**

Cardiovascular diseases are widespread across the globe, and heart failure (HF) accounts for the majority of heart-associated deaths. Target-based drug therapy is much needed for the management of heart failure. We have designed this study to evaluate icariin for its cardioprotective activity in the isoproterenol (ISO) induced postinfarction model. We have randomly distributed Wistar rats into seven groups, i.e., vehicle control; isoproterenol-treated; icariin per se; sildenafil per se; ISO + icariin 5; ISO + icariin 10; and ISO + sildenafil groups. ISO (85 mg/kg, subcutaneous) was administered at 24 hr for two consecutive days to produce cardiac injury, followed by icariin administration at 5 mg/kg and 10 mg/kg orally for 56 days.

**Materials and Methods:**

Rats were subjected to hemodynamic measurements biweekly. After 24 hr of the completion of dosing, animals were sacrificed, and markers for oxidative stress, fibrosis, inflammation, and cell death were measured. Transmission electron microscopy (TEM), histopathology, and MT staining of cardiac tissue were also done to assess the pathological and fibrotic architectural damage.

**Results:**

A significant decline in hemodynamics and an anti-oxidant collapse were found in ISO-intoxicated rats. Alterations in the levels of cyclic guanosine monophosphate (cGMP), interleukin-10 (IL-10), Tumor necrosis factor (TNF-α), and brain natriuretic peptide (BNP) were also observed in serum. Up-regulation of caspase-3, nuclear factor (NF-ĸB), and decline in expression of nuclear factor (NrF-2) contribute to cardiac damage. The treatment with icariin and sildenafil considerably reversed the toxic changes toward normal.

**Conclusion:**

Increased cGMP and Nrf2 expression and suppressed NF-ĸB-caspase-3 signaling play a pivotal role in icariin-mediated cardioprotection.

## Introduction

Globally, heart failure (HF) accounts for a vast number of hospital admissions and deaths. It imposes a high economic burden on patients and society (1). Despite significant advances in medical science, HF still claims productive lives worldwide. Hence, new strategies and treatments are always needed to manage heart failure better. 

Oxidative stress has a substantial role in the pathophysiology of HF. It is characterized by a persistent imbalance between producing and removing reactive oxygen species (ROS). Excessive ROS causes widespread damage to proteins, lipids, and DNA, resulting in abnormal cardiac function.

Furthermore, ROS contributes to developing infarction, fibrosis, remodeling, cardiac hypertrophy, and heart failure. Augmented ROS also induces different intracellular signaling pathways, evoking necrosis and apoptosis. So, the regulation and modulation of oxidative stress-mediated signaling pathways may prevent or delay the development of heart failure (2).

Epimedium herb is commonly used in treating coronary heart diseases, osteoporosis, impotence, and depression in traditional Chinese medicine (3, 4). Icariin, a chief constituent of Herba *epimedium*, is reported to have aphrodisiac, antidepressant, anti-tumor, immunomodulatory, osteoprotective, and cardioprotective properties (5–9). Song *et al*., performed a treatment study with icariin in isoproterenol-induced heart failure. They proposed that icariin successfully mediated the prevention of cardiac remodulation by inhibiting matrix metalloproteinases (9). Previous studies have shown that icariin can facilitate the growth of murine pluripotent stem cells into beating myocardiocytes (10). Additionally, our literature review indicated that icariin possesses anti-oxidant properties and can normalize oxidative stress in animal models (11–13).  

Therefore, we designed this study to assess the role of icariin in the isoproterenol-induced oxidative stress postinfarction model in rodents. This study targeted the hemodynamics, hypertrophy, ROS generation, and intracellular mechanisms involved in inflammation, programmed cell death, and heart failure. 

## Materials and Methods

***Animals***

After the approval of the Institutional Animal Ethics Committee (IAEC), Jamia Hamdard (Protocol number 1393, Approval date: 13/09/2017), we have procured male Wistar rats (200–250 g) from the Central Animal House Facility (CAHF), Jamia Hamdard, New Delhi (India). The animals were housed as per the guidelines of the Committee for the Purpose of Control and Supervision of Experiments on Animals (CPCSEA), Government of India. 

***Drugs and reagents***

Isoproterenol and icariin were procured from TCI Chemicals, India, and Cayman Chemicals, USA. The BNP and cGMP ELISA kits were purchased from Abcam, USA, and Arbor assays, USA. BD Biosciences, India, provided the cytometric bead array multiplex kits for TNF- and IL-10. The antibodies for NF-ĸB p65, NOS, Nrf2, and cleaved Caspase-3 immunohistochemistry were purchased from Santa Cruz Biotechnology. Analytical-grade chemicals were used in all the experimental work. All biochemical analyses were performed using HPLC-grade water.

***Experimental design***

*Induction of heart failure*

Heart failure was induced by administering isoproterenol (85 mg/kg, subcutaneous) in two consecutive doses at an interval of 24 hr (14). 

*Treatment groups and treatment schedule*

The treatment schedule for the study was as follows:

*Vehicle control group: *Received 0.1 ml 0.9% saline subcutaneously for 1^st^ and 2^nd^ of the study and vehicle DMSO (0.1%) in phosphate buffer solution orally for 56 days.

*Isoproterenol treated group: *Received 85 mg/kg isoproterenol in 0.9% saline subcutaneously for 1^st^ and 2^nd^ of the study and administered vehicle DMSO (0.1%) in phosphate buffer solution orally for 56 days.

*Icariin and Sildenafil per se: *Received 10 mg/kg icariin orally and 0.7 mg/kg sildenafil intraperitoneally, respectively, for 56 days.

*ISO + icariin 5 group: *Received 85 mg/kg isoproterenol in 0.9% saline subcutaneously for 1^st^ and 2^nd^ of the study and 5 mg/kg icariin orally for 56 days.

*ISO + icariin 10 group: *Received 85 mg/kg isoproterenol in 0.9% saline subcutaneously for 1^st^ and 2^nd^ of the study and 10 mg/kg icariin orally for 56 days.

*ISO + Sildenafil group: *Received 85 mg/kg isoproterenol in 0.9 % saline subcutaneously for 1^st^ and 2^nd^ of the study and sildenafil 0.7 mg/kg, intraperitoneally for 56 days.

At the termination of the study, MAP and HR were measured. Afterward, animals were sacrificed, and blood and heart samples were collected. Heart samples were soaked between filter paper to remove excess fluid. The heart samples were weighed and normalized to body weight. The tibia of the rat was also excised for length measurement. Small heart sections were preserved in 10% formalin for hematoxylin and Eosin staining and immunohistochemistry studies. Two millimeter pieces of heart tissue were preserved in Karnovsky’s reagent for electron microscopy. 

***In-silico*** ***study***

To better understand the binding mode of icariin at the molecular level, we carried out molecular docking simulations of icariin at TNF-α, Nrf2, and NF-κB protein receptor catalytic ligand binding sites (PDB ID: 5MU8, 6FFM, and 1VKX, respectively). The docking of icariin was performed using Maestro, version 10.6, implemented from the Schrodinger software suite. The ligands were sketched in a 3D format using a build panel and were prepared for docking using the ligprep application. The proteins for the docking study were taken from the protein data bank (PDB ID: 5MU8, 6FFM, and 1VKX) and prepared by removing the solvent, adding hydrogen, and further energy minimization using the protein preparation wizard. Grids for molecular docking were generated with the standard ligand, sildenafil. Icariin was docked using the Glide extra-precision (XP) mode, and each molecule could save up to three poses.

***Non-invasive blood pressure measurement***

MAP and HR of rats were recorded by a tail-cuff method using a non-invasive BP instrument. The rats were trained daily in a restrainer for 15 min for one week. MAP and HR were calculated using a computerized data acquisition system. MAP and HR were recorded at 0 days (baseline), 14^th^, 28^th^, 42^nd^, and 56^th^ days of the treatment schedule. 

***Biochemical estimations in myocardial tissue***

The levels of thiobarbituric acid reactive substances (TBARS), superoxide dismutase (SOD), catalase (CAT), and reduced glutathione (GSH) were assessed using methods published earlier (15-18).

***Estimation of nitric oxide***

The nitrite accumulation in the myocardial tissue indicates nitric oxide production (NO). Colorimetric assay with Griess reagent was used to calculate nitric oxide concentration. Tissue homogenate supernatant and Griess reagent were mixed in equal volumes. We kept this mixture at room temperature for 10 min, and absorbance was recorded at 540 nm. The sodium nitrite standard curve quantitively estimated the nitrite level in the supernatant.

***Measurement of cGMP and BNP***

The cGMP level and BNP levels were quantified in myocardial tissue and serum using an ELISA kit following the manufacturer’s instructions. 

***Quantification of TNF-α and IL-10***

The concentrations of TNF–α and IL–10 in serum were determined by cytometric bead array technology (BD Biosciences, San Diego, California) and fluorescence detection by flow cytometry as per the manufacturer’s recommendations. 

***Histology***

For histologic examination, heart sections kept in formalin were sectioned transversely and embedded in paraffin. These sections were stained with hematoxylin and eosin dye to assess histopathological changes. Images were taken at 10X and 40X by a Meiji microscope enabled with infinity analysis software.

***Transmission electron microscopy (TEM)***

The samples kept in modified Karnovasky’s fluid were further buffered with 0.1 M sodium phosphate for 15 hr. These sections were later fixed with 1% osmium tetroxide (buffer 0.1 M sodium phosphate) for two hours at 4 °C. After washing with 0.1 N sodium phosphate buffers, the specimens were dehydrated in graded acetone solutions and embedded in CY212. Then, ultrathin sections (60–80 nm) were stained with alcoholic acetone and lead citrate and examined with the transmission electron microscope TECNAI 200 kV TEM (Fei, electron optics, Hillsboro, USA).

***TUNEL assay***

TUNEL assay was used to assess DNA fragmentation, a significant event of apoptosis (19), by incorporating FITC–dUTP catalytically at the 3’-hydroxy end of the fragmented DNA as per the manufacturer’s instructions (Apo direct Kit, Roche). The single-cell suspension was prepared from cardiac tissue (20). Cells were washed twice using phosphate buffer and then suspended in 0.2 ml phosphate buffer solution. Later, it was fixed by adding 1 ml of 2% paraformaldehyde on ice for one hour. Samples were washed with phosphate buffer solution and incubated with 3% hydrogen peroxide in methanol for 10 min at 25 °C, followed by washing with phosphate buffer solution.

Further, these cells were permeabilized with freshly prepared chilled 0.1% Triton X-100 for 5 min on ice. The cells were washed twice with PBS, after which 50 ml of a reaction mixture containing TdT and FLUOS-labelled dUTP was added for one hour at 37 °C. The cells were washed and suspended in PBS for data acquisition on a BD LSR II flow cytometer and analyzed using Deva software. Histogram analysis of FL-1H (x-axis; FITC fluorescence) was recorded to show the shift in fluorescence intensity compared with the control.

***Immunohistochemistry of myocardial tissue***

Paraffin sections (5 µm thick) were deparaffinized in xylene, rehydrated with a graded ethanol series, and washed under running double distilled water. Sections were subjected to antigen retrieval using citrate buffer (pH 6). They were allowed to cool at room temperature for 10 min, then incubated in 4% hydrogen peroxide for 15 min to remove the background staining. Sections were washed three times using TBS and were incubated overnight at 4 °C with the primary antibody. They were then rinsed with buffer and incubated for one hour with a peroxidase-conjugated secondary antibody. The reaction was visualized using a diaminobenzidine solution. Photomicrographs were taken with a computer-enabled Meiji microscope. The Fiji (Image J) software was used to semi-quantify the protein expression using the reciprocal intensity method. The image pixel intensities ranged between 0 and 250. Values 0 and 250 indicate the darkest and the lightest shade of image color, respectively.

***Data presentation and statistical analysis***

GraphPad 5.0 was used for statistical analysis (GraphPad Software; San Diego, CA, USA). All values in the tables and figures are expressed as mean±SEM. ‘n’ represents the number of animals studied. The results were analyzed using one-way ANOVA, followed by a *post hoc* comparison using Tukey’s multiple comparisons test. A Repeated-measures ANOVA was used to analyze the change in each group, followed by a *post hoc* comparison using the Bonferroni multiple comparison tests. A *P*-value less than 0.05 was considered significant. 

## Results

***Molecular docking studies***

We have assessed the* in silico* binding of icariin against TNF-α, Nrf2, and NF-κB p65 protein receptors, and the results revealed a standard binding orientation of icariin in the binding pocket of TNF-α, Nrf2, and NF-κB p65 protein receptors (PDB ID: 5MU8, 6FFM, and 1VKX). We reported the binding position of icariin with the respective proteins and compared it with sildenafil. Icariin interacted with the TNF-α protein receptor’s backbone at Tyr119, Gly121, and Tyr151. Icariin superimposition with sildenafil demonstrated the identical interaction observed in the TNF-protein receptor’s catalytic domain and a higher docking score than the standard ligand (sildenafil). Icariin formed hydrogen bonds with the backbone of the Nrf2 protein receptor at Asp87 and His154. 

**Figure 1:**
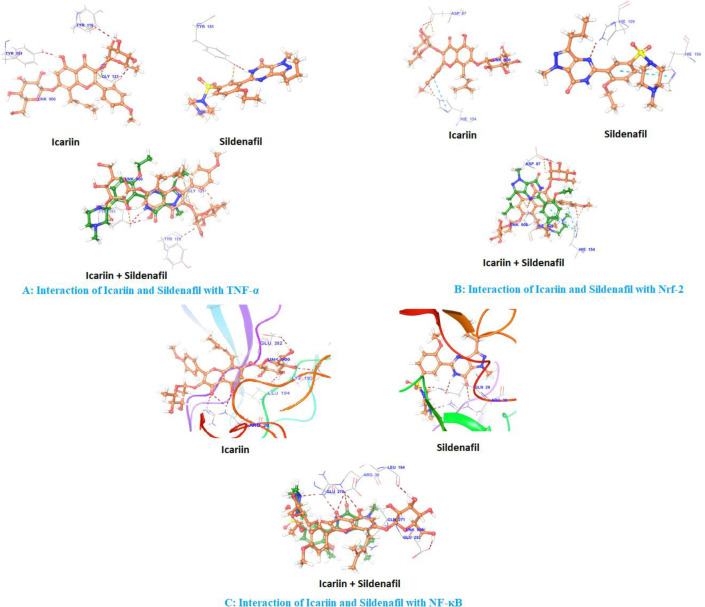
*In silico* analysis of icariin and sildenafil

Further, this compound also showed pi-pi stacking with His154. Icariin superimposition with sildenafil showed the identical interaction observed in the catalytic domain of the Nrf2 protein receptor and showed a better docking score than the standard ligand (sildenafil). Icariin interacted with the backbone of the NF-κB protein receptor at Arg30, Leu194, Lys195, and Glu282. Icariin superimposition with sildenafil showed a similar interaction observed in the catalytic domain of the NF-κB protein receptor and showed a better docking score than the standard ligand (sildenafil) (Figure 1).

***Anthropometric parameters***

Treatment with isoproterenol causes substantial weight loss compared with the control group (*P*<0.01). However, icariin (10 mg/kg) and sildenafil treatment prevented this weight loss in comparison with the isoproterenol-treated group (*P*<0.001). The gravimetric assessment showed that heart weight (g), heart weight/body weight ratio (mg/g), and heart weight/tibial length ratio (mg/mm) considerably increased with the isoproterenol treatment (*P*<0.001). Icariin and sildenafil administration prevented the unfavorable changes in isoproterenol-treated groups (*P*<0.01 *vs* icariin 5; *P*<0.001 *vs* icariin 10 & sildenafil) (Table 1).

***Hemodynamic parameters***

**Figure 2:**
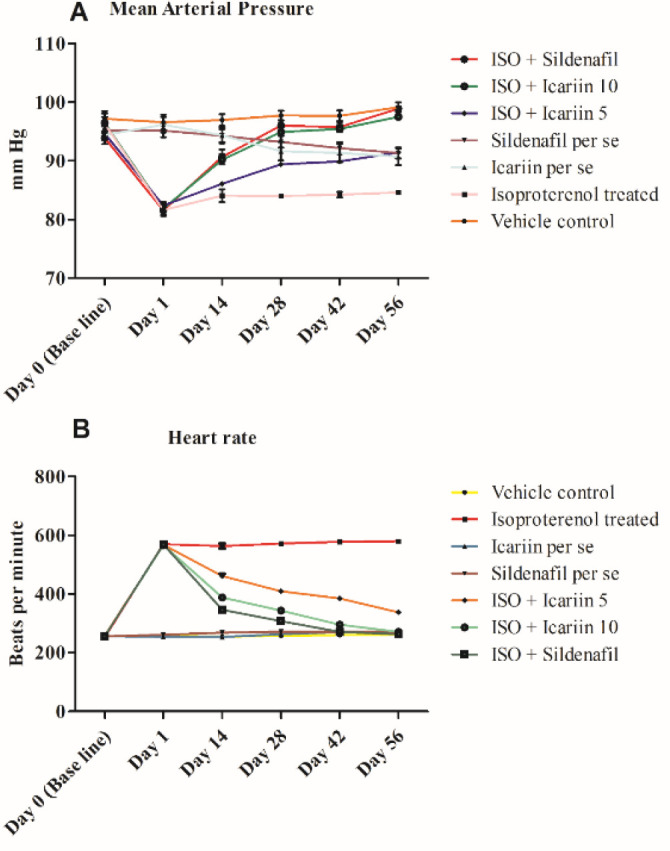
Hemodynamic measurement in different groups across the timeline of the study

At baseline, no substantial differences were observed in the different groups. After isoproterenol administration, except *per se* groups, all other groups showed a substantial fall in mean arterial pressure (*P*<0.001). At day 14, treatment with icariin at the dose of 10 mg/kg and sildenafil considerably reversed the damage done by isoproterenol (*P*<0.001). However, low-dose icariin elevated the arterial pressure non-significantly. At days 28, 42, and 56, icariin (both 5 & 10 mg/kg) and standard sildenafil significantly ameliorated the fall in mean arterial pressure (*P*<0.001) (Figure 2A). 

After the administration of isoproterenol, the heart rate of ISO-treated groups rose markedly (*P*<0.001). Treatment with icariin (both doses) and sildenafil substantially reduced heart rates to normal. They were comparable to the vehicle control group (Figure 2B).

***Endogenous glutathione and anti-oxidant enzyme***

**Figure 3:**
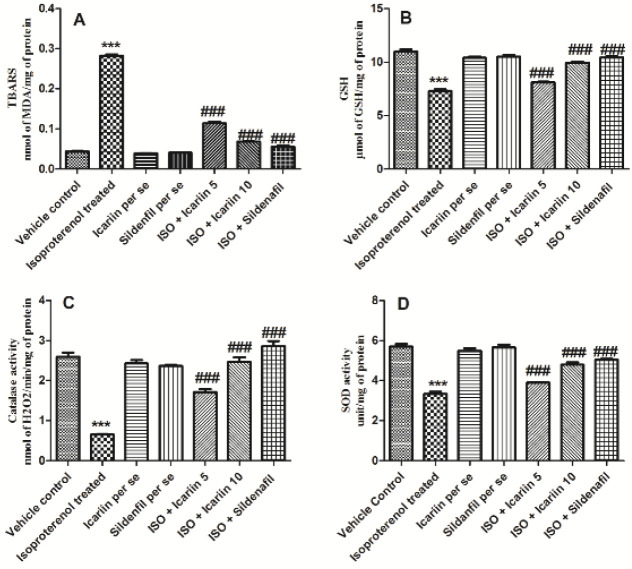
Status of anitoxidant defence in different groups of isoproterenol treated rats. (A) TBARS, (B) GSH, (C) Catalase, (D) SOD. Values = Mean ± Standard error of mean (n=6). ***P<0.001 vs Vehicle control; ###*P*<0.001 vs isoproterenol treated

Icariin and sildenafil maintained the anti-oxidant defense weakened by isoproterenol treatment. After subcutaneous administration of isoproterenol, an amplified level of MDA and a decline in SOD, Catalase, and GSH levels were observed (*P*<0.001). The treatment with icariin and sildenafil considerably reversed the collapse in anti-oxidant defense induced by isoproterenol (*P*<0.001) (Figure 3). 

***Pro- and anti-inflammatory cytokines***

**Figure 4:**
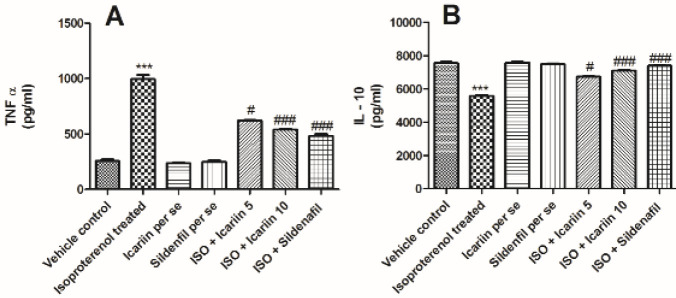
Cytokine levels in different groups of isoproterenol treated rats (A) TNF α, (B) IL -10. Values = Mean ± Standard error of mean (n=6). ***P< 0.001 vs Vehicle control; #*P*<0.05 vs isoproterenol treated; ###*P*< 0.001 vs isoproterenol treated

Figure 4 depicts the variations in TNF- and IL-10 levels in different treatment groups. ISO administration considerably elevated the TNF–α and reduced the IL-10 level compared with the vehicle control group (*P*<0.001). Icariin (5 mg/kg & 10 mg/kg) and sildenafil treatment elevated IL-10 levels compared with the toxic group. Additionally, icariin treatment (5 mg/kg and 10 mg/kg) considerably reduced the TNF-α level (*P*<0.001). Similarly, sildenafil reduced the TNF-α level (*P*<0.001) compared with the isoproterenol-treated group. 

***cGMP, NO, and BNP levels***

Table 2 shows that isoproterenol treatment disrupted cellular integrity and induced inflammatory response. These changes were represented by a substantial rise in NO and BNP levels (*P*<0.001). A slight increase in cGMP also occurred with the administration of isoproterenol (*P*<0.05). Icariin and sildenafil treatment considerably increased the cGMP level compared with the toxic group (*P*<0.001). However, the levels of NO and BNP decreased with the administration of icariin (*P*<0.01 with icariin at 5 mg/kg; *P*<0.001 with icariin at 10 mg/kg) and sildenafil (*P*<0.001). 

***Histopathology***

**Figure 5:**
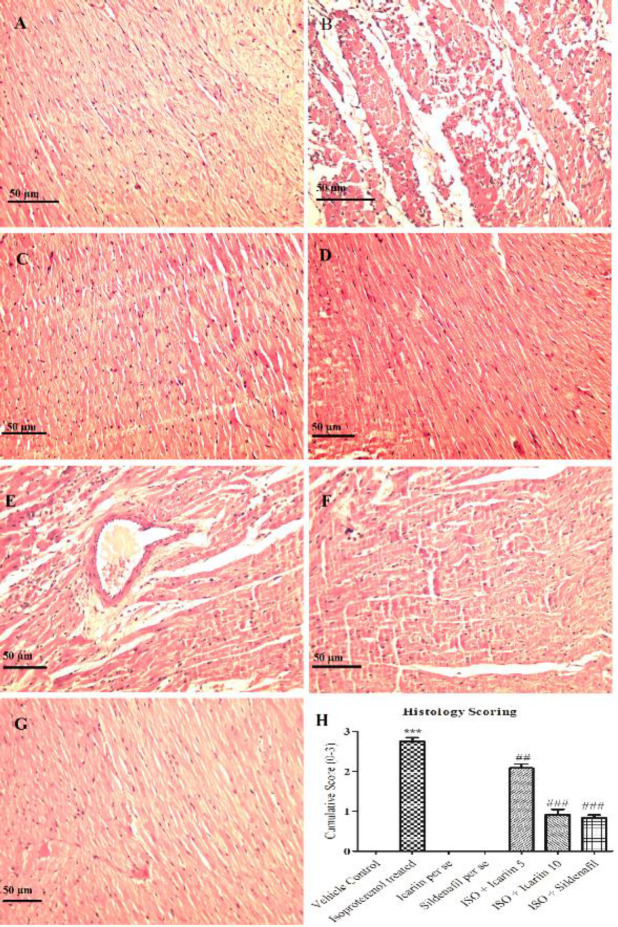
Hematoxylin and and eosin staining in heart tissues of various groups (10X) [Scale bar - 50 μm]. (A) Vehicle control, (B) Isoproterenol treated, (C) Icariin per se, (D) Sildenafil per se, (E) ISO + icariin 5, (F) ISO + icariin 10, (G) ISO + Sildenafil (H) Bar graph showing histological scoring. ***P<0.001 vs Vehicle control; ##*P*<0.01 vs isoproterenol treated; ###*P*<0.001 vs isoproterenol treated

Figure 5 represents the histopathological alterations in different groups. Vehicle control, icariin *per se*, and sildenafil *per se* displayed the normal integrity of heart tissue with branched appearance, striations, and continuity in myofibrils. The Isoproterenol-treated group exhibited substantial myocyte degeneration, cardiac cell death, infiltration of lymphocytes and macrophages, and misshapen cell nuclei. Treatment with icariin at 10 mg/kg and sildenafil reversed the necrotic changes and cellular infiltration. Another dose of icariin (5 mg/kg/day) has shown less protection than the higher dose of icariin and sildenafil.

***Transmission electron microscopy (TEM)***

**Figure 6:**
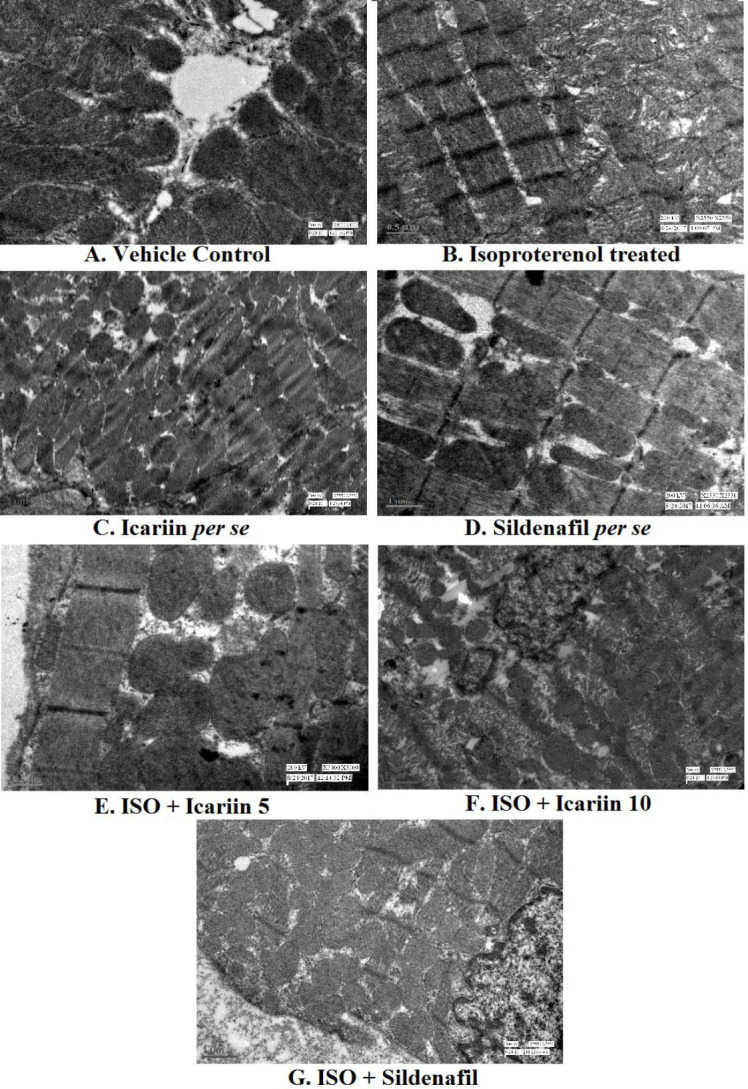
TEM of myocardial tissue of different groups of isoproterenol treated rats (A) Vehicle control, (B) Isoproterenol treated, (C) Icariin per se, (D) Sildenafil per se, (E) ISO + icariin 5, (F) ISO + icariin 10, (G) ISO + Sildenafil (H). ISO – Isoproterenol

Figure 6 displays the micrographs of cardiac tissue in different groups. The micrographs of vehicle control and *per se* groups presented a standard ultrastructural shape of myofibrils with sharp Z bands set in regular rows and mitochondria between the myofibrils. The myocytes of the isoproterenol-treated group presented ultrastructural damage with loss of myofibrils, moderate mitochondrial swelling, and condensed chromatin in the nucleus. The micrographs of icariin and sildenafil-treated groups showed intact myofibrils with mild mitochondrial swelling and prevented chromatin condensation.

***DNA fragmentation using TUNEL***

**Figure 7:**
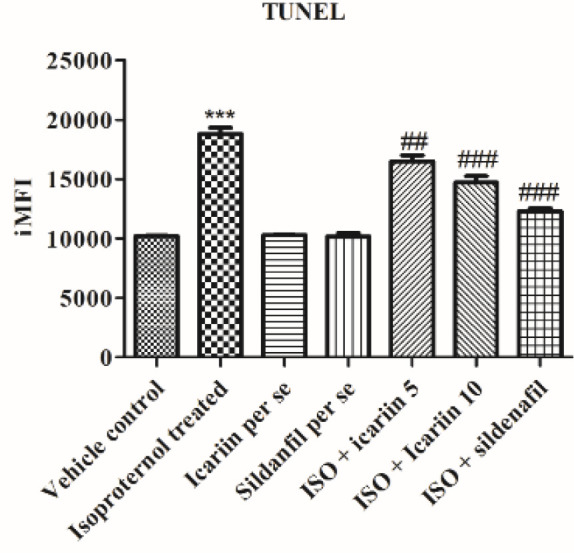
Quantification of DNA Fragmentation in different groups. Values = Mean ± Standard error of mean (n=6). ***P<0.001 vs Vehicle control; ##*P*<0.01 vs isoproterenol treated; ###*P*<0.001 vs isoproterenol treated. ISO: Isoproterenol, Terminal deoxyribonucleotidyltransferase (TdT)-mediated dUTP nick-end labeling

dTP-FLOUS labeling, in terms of iMFI, was used to quantify DNA fragmentation. Figure 7 shows a rise in iMFI in the isoproterenol-treated group compared with the control group (*P*<0.001), providing substantial evidence for apoptosis. *Per se* groups showed a non-significant rise in iMFI. However, icariin and sildenafil-treated groups exhibited a significant fall in iMFI in comparison with the isoproterenol-treated group (*P*<0.001).

***Nrf2, Caspase-3, and NF – ĸB expression levels***

**Figure 8:**
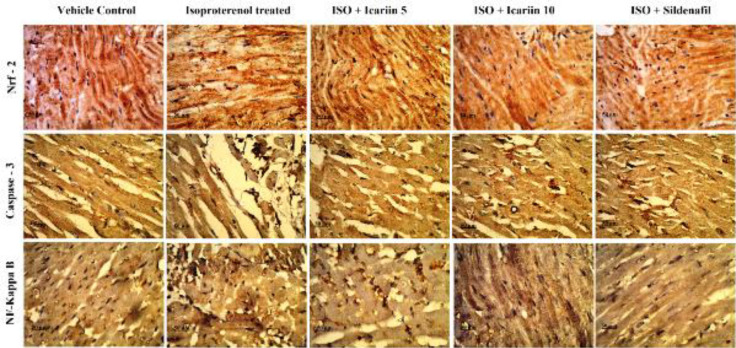
IHC of Nrf2, Caspase-3 and NF- ĸB in various group of isoproterenol treated rats [Scale bar - 50 μm]

ISO: Isoproterenol; Nrf2: Nuclear factor (erythroid derived 2) - like 2; NF-ĸB: Nuclear factor-ĸB

**Figure 9:**
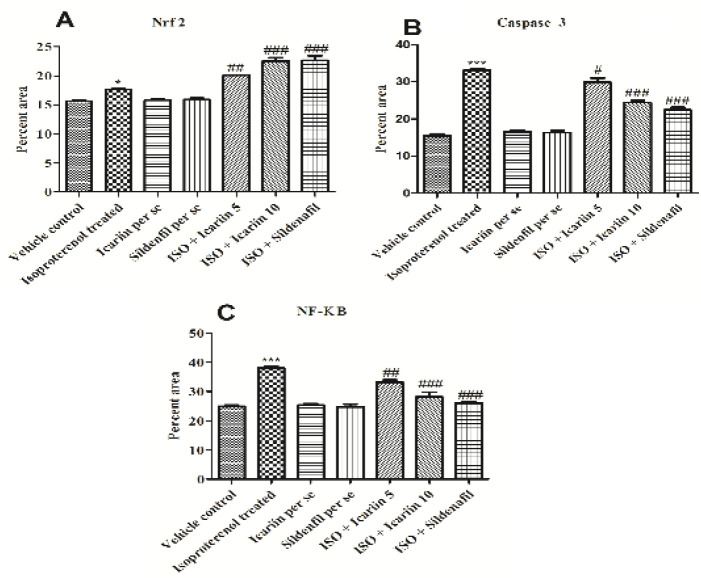
Semi quantitative evaluation of IHC among different groups of isoproterenol treated rats (A) Nrf2, (B) Caspase-3 and (C) NF- ĸB. Values = Mean ± Standard error of mean (n=6). ***P<0.001 vs Vehicle control; *P<0.5 vs Vehicle control; ##*P*< 0.01 vs isoproterenol treated; #*P*<0.5 vs isoproterenol treated; ##*P*<0.01 vs isoproterenol treated; ###*P*<0.001 vs isoproterenol treated

ISO: Isoproterenol; Nrf2: Nuclear factor (erythroid derived 2)-like 2; NF; ĸB- Nuclear factor-ĸB

Figures 8 and 9 show variations in the expression of Nrf2, Caspase-3, and NF – ĸB in various groups. The treatment with icariin (5 mg/kg and 10 mg/kg) and sildenafil (0.7 mg/kg) decreased the expression of NF–ĸB and cleaved Caspase-3. On the other hand, the expression of Nrf2 was elevated with the treatment of icariin and sildenafil. 

***Cardiac fibrosis using TGF1-β1 and MT stain***

**Figure 10:**
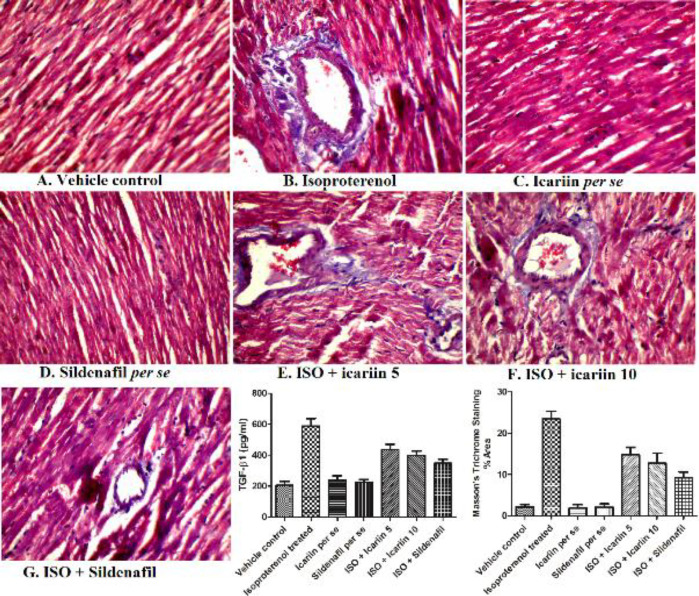
Cardiac fibrosis and TGF-β1 level using Masson’s trichome statining in different isoproterenol treated rats. Values = Mean ± Standard error of mean (n=6).

Figure 10 shows the increased myocardial fibrosis upon isoproterenol administration, as estimated by estimation of TGF1-β1 and MT stain. However, treatment with icariin at the doses of 5 mg/kg and 10 mg/kg and sildenafil at 0.7 mg/kg considerably reduced fibrosis in a dose-dependent manner. 

## Discussion

This study evaluated icariin’s cardioprotective effect on the experimental heart failure model in Wistar rats. Isoproterenol administration stimulates oxidative stress in the heart, which is critical in producing cardiac injury (21). Icariin, a polyphenol from the *epimedium* herb, has been traditionally used as a cardioprotective, osteoprotective, and nephroprotective agent for centuries (3). Earlier studies on icariin have demonstrated its anti-oxidant activity and role in preventing cardiac remodeling after isoproterenol exposure in rats (9, 13). However, the exact mechanism of cardioprotection by icariin is still unpredictable. 

Isoproterenol at supramaximal concentration causes morphological and physiological changes in the heart. It further leads to loss of anti-oxidant defense, cellular injury, decreased diastolic and systolic function, hyperplasia, and heart failure, closely resembling the post-myocardial infarction pathological changes observed in humans (14, 22-24). Auto oxidation of isoproterenol causes an upsurge in the production of cytotoxic free radicals, which plays a crucial role in isoproterenol-induced myocardial damage. The production of free radicals causes the peroxidation of membrane phospholipids, which changes membrane permeability and increases intracellular calcium overload (21). 

We saw a significant rise in heart weight, heart weight/tibial length, and heart weight/body weight ratio in isoproterenol-treated rats. Previous studies have already established that increased heart weight with isoproterenol treatment is observed because of fluid retention, inflamed intramuscular space, and extensive cardiac cell death followed by infiltration of inflammatory cells (25). In isoproterenol-intoxicated rats, treatment with icariin and sildenafil considerably decreased the heart weight and its ratio with body weight and tibial length. Alteration in systolic and diastolic function and increased heart rate in isoproterenol-treated rats is another characteristic feature of physiological loss of heart activity (26). 

We noted a considerable decline in mean arterial blood pressure and an increase in heart rate after isoproterenol administration. The observed changes in arterial pressure might be due to myocardial necrosis, leading to lower cardiac output, forcing the heart to match up with the body’s needs and increasing the heart rate (27). Treatment with icariin inhibited the loss of systolic and diastolic function, thereby suggesting its cell membrane protective activity. 

Lipid peroxidation occurs due to the oxidative deterioration of polyunsaturated lipids. Catecholamine-derived free radicals can react with lipids to produce lipid peroxides which can do significant injury (21). Lipid peroxidation increases membrane permeability and makes it more vulnerable to oxidative damage. This study showed elevated MDA levels, which indicated extensive lipid peroxidation. These changes were significantly reversed by treatment with icariin and sildenafil.

Endogenous reduced glutathione is a tripeptide. It shows its anti-oxidant function by attaching to the superoxide radicals, singlet oxygen radicals, and peroxy radicals to produce oxidized glutathione. GSH regulates several cellular processes and protects cells from free radical-mediated damage (28). The decline in GSH levels is likely associated with enhanced oxidative stress in cardiac injury (21). This study observed a decrease in endogenous GSH levels, further normalized by the treatment with icariin and sildenafil. Our results showed the anti-lipid peroxidative effects of icariin and sildenafil against damage caused by free radicals. Previously published literature on icariin and sildenafil in different models showed marked elevation in GSH and a decline in MDA levels. (29). 

Free radical scavenging enzymes like SOD and catalase are the first lines of anti-oxidant defense. The equilibrium between the free radical scavenging enzymes and oxidative stress is crucial for maintaining homeostasis. Isoproterenol’s auto-oxidation generates quinones that produce superoxide anions and hydrogen peroxide by interacting with oxygen. They collectively create immense oxidative stress and deplete endogenous anti-oxidant enzymes. Thus, administration of isoproterenol disrupts this equilibrium with an increased demand for anti-oxidant enzymes (30). Our study demonstrated a significant lowering of SOD and catalase after isoproterenol administration which is in line with several previous studies. The fall in the activity of SOD and catalase might be due to their increased utilization for scavenging reactive oxygen moieties (21). The treatment with icariin and sildenafil considerably prevented the drop in the level of SOD and catalase in comparison with isoproterenol-treated groups and thus suggested their anti-oxidant property. 

The role of nitric oxide is very controversial in the physiology of the heart. Conventionally, nitric oxide is an essential vasodilator produced by coronary endothelial cells and exerts cardioprotective action. It serves as a free radical scavenger and minimizes the harmful effects of oxidative stress (31). However, several studies reported the other side of the story. They showed that increased nitric oxide production by iNOS might contribute to the negative inotropic effect and severe inflammatory response by activating NF-ĸB (32). Many favorable nitric oxide signals are mediated by cGMP, produced by soluble guanylate cyclase after activation of nitric oxide. In cardiomyocytes, cGMP is degraded by PDE–5 enzymes, so the inhibition of PDE–5 enzymes is expected to protect the heart by increasing the myocardial level of cGMP (33). 

Our study involved the treatment of isoproterenol-intoxicated rats with icariin and sildenafil; both are PDE-5 inhibitors, thus contributing to the rise in cGMP levels in heart tissue. After isoproterenol treatment, nitric oxide levels rose abruptly, but treatment with icariin and sildenafil lowered their concentrations compared with the isoproterenol-treated group. Therefore, we assume that activation of NO/cGMP axes is one of the likely mechanisms for icariin-mediated cardioprotection.

BNP is a peptide hormone mainly released by cardiomyocytes in the ventricles in response to stretching caused by a rise in ventricular volume. Measuring its plasma concentration has the clinical significance of diagnosing heart failure and excluding other conditions like dyspnoea or fluid retention. A literature survey showed cardiac injury caused by isoproterenol leads to increased BNP levels (34). We observed a substantial rise in BNP levels after isoproterenol administration compared with the vehicle control group; however, treatment with icariin and sildenafil significantly decreased the BNP levels compared with isoproterenol-treated rats. These results align with a previous study that showed that treating CHF rats with icariin led to a dose-dependent decline in BNP levels (9). 

It is well-documented that treatment with isoproterenol causes necrosis and apoptosis in rats’ hearts. Administration of isoproterenol activates the pro-apoptotic protein, Bax, which further causes permeabilization and damage to the mitochondrial membrane and activates effectors called caspases. However, the antiapoptotic protein, Bcl-2 protein, inhibits this cascade. Initially, caspase-9 gets activated with the release of cytochrome c by Bax, which later in the process activates caspase-3 and concludes the apoptosis via different events such as DNA fragmentation, chromatin condensation, and poly-ADP-ribose polymerase cleavage (35,36). Isoproterenol administration considerably increased the expression of caspase-3 in cardiac tissue and DNA fragmentation in the cardiac cell. Afterward, treatment with icariin and sildenafil attenuated the expression of caspase-3 and inhibited the apoptotic cascade. These observations indicated that icariin efficiently attenuated the apoptosis induced by ISO, and these results were well correlated with the hemodynamic and anti-oxidative enzyme activities.

In our study, ISO administration resulted in NF–ĸB overexpression, which mediated the movement of pro- (TNF–α) and anti- (IL–10) inflammatory cytokines. Similar results have been obtained with previous studies, which showed the role of iNOS, NF–ĸB, and cytokines in the progression of heart failure (36,37). The treatment with icariin and sildenafil significantly suppressed NF–ĸB expression. The altered IL–10 and TNF–α have also returned to normal after treatment with icariin and sildenafil.

Previous studies showed the changes in the transcriptional regulation of anti-oxidant genes in experimental disease models (25, 35). Nrf2 is a crucial transcriptional regulator of endogenous anti-oxidants like SOD, catalase, and other GSH-regulated enzymes (25). Some earlier studies reported that Nrf2 was pivotal in preventing isoproterenol-induced cardiac cell damage and related events like apoptosis and heart failure (38). We found a significant rise in the levels of Nrf2 in the infarct border zone after isoproterenol treatment, which was further enhanced by treatment with icariin and sildenafil. Similarly, isoproterenol administration caused myocardial fibrosis and increased the expression of collagen and level of TGF-β1 and participated in the event of heart failure. However, treatment with icariin and sildenafil significantly reduced collagen expression and the level of TGF-β1 expression, thus exhibiting an anti-fibrotic effect. 

**Table 1:** Anthropometric parameters in isoproterenol-challenged rats

Group	Vehicle control	Isoproterenol treated	Icariin *per se*	Sildenafil *per se*	ISO + Icariin 5	ISO + Icariin 10	ISO + Sildenafil
Body weight (g)	360.8 ± 2.71	294.2 ± 4.90^***^	342.5 ± 8.53	351.7 ± 7.49	316.7 ± 2.47	325 ± 5.0^###^	329.2 ± 4.36^###^
Heart weight (mg)	701.7 ± 12.49	854.7 ± 9.68^***^	693.3 ± 7.6	699.2 ± 8.40	828.2 ± 12.54	791.7 ± 3.07^##^	726.7 ± 12.02^###^
Tibia length (mm)	46.17 ± 0.16	46.5 ± 0.22	45.5 ± 0.21	45.5 ± 0.20	46.50 ± 0.22	45.50 ± 0.22	45.83 ± 0.30
Heart weight/ Body weight (mg/g)	1.94 ± 0.04	2.91 ± 0.06^***^	2.03 ± 0.06	1.99 ± 0.03	2.61 ± 0.04^##^	2.43 ± 0.03^###^	2.20 ± 0.03^###^
Heart weight/Tibial length (mg/mm)	15.20 ± 0.27	20.54 ± 0.49^***^	15.24 ± 0.16	15.42 ± 0.2	17.77 ± 0.27^##^	16.62 ± 0.37^###^	15.86 ± 0.26^###^

Values = Mean ± Standard error of the mean (n=6). ***P<0.001 vs Vehicle control; ##*P*<0.01 vs isoproterenol treated; ###*P*<0.001 vs isoproterenol treated

ISO: Isoproterenol

TBARS: Thiobarbituric acid reactive substances, GSH: Reduced glutathione, SOD: Superoxide dismutase

**Table 2:** Levels of nitric oxide, cGMP and BNP in various groups of isoproterenol treated rats

Group	Vehicle control	Isoproterenol treated	Icariin *per se*	Sildenafil *per se*	ISO + Icariin 5	ISO + Icariin 10	ISO + Sildenafil
Nitrite	5.69 ± 0.19	9.53 ± 0.14^***^	6.01 ± 0.07	5.53 ± 0.15	8.4 ± 0.12^###^	7.88 ± 0.02^###^	7.4 ± 0.16^###^
cGMP	3.93 ± 0.22	3.15 ± 0.02^*^	8.42 ± 0.13	8.69 ± 0.18	10.26 ± 0.15^###^	11.42 ± 0.13^###^	12.63 ± 0.16^###^
BNP	2.91 ± 0.22	6.42 ± 0.08^***^	2.58 ± 0.21	3.10 ± 0.05	5.57 ± 0.11^##^	4.74 ± 0.18^###^	4.66 ± 0.11^###^

Values = Mean ± Standard error of mean (n=6). ***P<0.001 vs Vehicle control; *P<0.05 vs Vehicle control; ##*P*<0.01 vs isoproterenol treated; ###*P*<0.001 vs isoproterenol treated

cGMP: Cyclic Guonosine monophosphate; BNP: Brain natriuretic peptide

TNF α: Tumor necrosis factor α; IL– 10: Interleukin–10

ISO: Isoproterenol

***P<0.001 vs Vehicle control; ###*P*<0.001 vs isoproterenol treated

ISO: Isoproterenol; TGF-β1: Transforming growth factor beta 1

## Conclusion

Our study involved the isoproterenol-mediated oxidative stress-induced pathological changes that led to heart failure. Furthermore, treating intoxicated rats with icariin showed relief of various harmful changes like inflammation, anti-oxidant failure, hemodynamic loss, apoptosis, histopathological and ultrastructural changes, and fibrosis. The alteration of the NF-ҡB inflammatory pathway, caspase-3-induced DNA fragmentation, NO-cGMP pathway, and Nrf2 signaling activation is undeniable. Thus, considering all of the above parameters, we concluded that icariin in both doses acted as a cardioprotective agent and was quite comparable with the standard drug sildenafil. 

## Authors’ Contributions

SS, AI, VK, AKN, and SEH designed the experiments; SS performed experiments and collected data; KCS performed the *in silico* studies. SS, AI, and VK discussed the results and strategy; SEH supervised, directed, and managed the study; SS, AI, VK, KCS, AKN, and SEH approved the final version of the manuscript to be published. 

## Conflicts of Interest

The authors declare no conflicts of interest.
